# Screening of Predatory Natural Enemies of *Lygus pratensis* in Cotton Fields and Evaluation of Their Predatory Effects

**DOI:** 10.3390/insects16090903

**Published:** 2025-08-28

**Authors:** Pengfei Li, Kunyan Wang, Tailong Li, Liqiang Ma, Changqing Gou, Hongzu Feng

**Affiliations:** 1 Key Laboratory of Integrated Pest Management (IPM) of Xinjiang Production and Construction Corps in Southern Xinjiang, Tarim University, Alar 843300, China; 10757202064@stumail.taru.edu.cn (P.L.); 13164316063@163.com (K.W.); 18738725702@163.com (T.L.); 19326684915@163.com (L.M.); 2 Scientific Observing and Experimental Station of Crop Pests in Alar, Ministry of Agriculture, Tarim University, Alar 843300, China; 3 The National and Local Joint Engineering Laboratory of High Efficiency and Superior-Quality Cultivation and Fruit Deep Processing Technology on Characteristic Fruit Trees, Tarim University, Alar 843300, China; 4 Key Laboratory of Protection and Utilization of Biological Resources in Tarim Basin, Tarim University, Alar 843300, China

**Keywords:** mirid bug, generalist predators, molecular detection, predation function, biological control

## Abstract

*Lygus pratensis* is a major pest of cotton, causing serious damage to cotton production. This study designed species-specific PCR detection primers for *L. pratensis*, established a detection system to identify *L. pratensis* DNA in the intestinal contents of predatory natural enemies, and investigated the control potential of four species’ predatory natural enemies against *L. pratensis*. The results indicated that 826 predatory natural enemies were collected from cotton fields belonging to two classes, five orders, and twelve families. Among these, 9 species of insecta natural enemies accounted for 54.12% of the total number of predatory natural enemies collected, while 14 species of arachnida predatory natural enemies comprised 45.88%. Of the 806 natural enemies tested, 5.58% were found to be positive for *L. pratensis*, all of which were arachnid predators, specifically *Ebrechtella tricuspidata*, *Xysticus ephippiatus*, *Hylyphantes graminicola*, and *Oxyopes sertatus*. The predation response of these four spider species to the fourth to fifth instar nymphs and adults of *L. pratensis* adhered to the Holling II model. The theoretical predation (*a′*/*Th*), daily maximum predation rate (*T*/*Th*), and searching effect for the fourth to fifth instar nymphs and adults of *L. pratensis* of the four spider species were assessed. According to the results, the species can be ranked in terms of their predatory and searching efficiency as follows: *O. sertatus* > *E. tricuspidata* > *X. ephippiatus* > *H. graminicola*. Four species of spiders had the highest theoretical predation against *L. pratensis* nymphs, ranging from 23.71 to 60.86, and adults, ranging from 22.14 to 50.25. Therefore, these four spider species could be utilized for *L. pratensis* management. This study identified the main predatory natural enemies of *L. pratensis* and their pest control capabilities, providing a scientific basis for selecting and utilizing natural enemies in integrated pest management (IPM) strategies. This will help promote ecological and green pest control of *L. pratensis* in cotton-growing areas.

## Simple Summary

Cotton is a crucial natural fiber source worldwide. *Lygus pratensis* is one of the primary pests of cotton, causing serious damage to cotton production. The control of *L. pratensis* has long relied on chemical pesticides, leading to increased resistance and environmental pollution. Using natural enemies in pest control offers new ideas for exploring effective and sustainable methods to control the damage caused by *L. pratensis*. In this study, we designed species-specific primers for *L. pratensis*. The species of natural enemies targeting *L. pratensis* were identified by measuring the DNA of *L. pratensis* in the intestinal contents of predatory natural enemies. Four spider species’ functional responses and control potential against *L. pratensis* were preliminarily evaluated, providing a basis for spiders as natural enemies of *L. pratensis*. The results indicated that *Oxyopes sertatus*, *Ebrechtella tricuspidata*, *Xysticus ephippiatus*, and *Hylyphantes graminicola* had the highest theoretical predation against *L. pratensis* nymphs, ranging from 23.71 to 60.86, and adults ranging from 22.14 to 50.25, showing good control potential against *L. pratensis*.

## 1. Introduction

Cotton is an essential source of natural fiber and plays a significant role in global fiber production [1,2]. Cotton is affected by various pests during production, causing severe economic losses to cotton production [3]. *Lygus pratensis* (Linnaeus, 1758) (Hemiptera: Miridae) is a significant agricultural pest extensively reported in Europe, North Africa, the Middle East, India, and China [4,5]. This pest causes considerable damage to cotton production [6,7]. They mainly damage cotton with their piercing-sucking mouthparts, resulting in wilting and deformation of buds and leaves, as well as shedding of petals. In severe cases, the dropping of flower buds and bolls even leads to the premature death of the cotton [8,9,10]. Since the mid-1990s, with the widespread cultivation of transgenic *Bacillus thuringiensis* (Bt) cotton globally [11], the use of broad-spectrum insecticides in cotton fields has diminished [12,13], with the absence of effective control measures for *L. pratensis* [14,15]. Recently, the proportion of forest belts and building land has increased. These non-crop habitats provide *L. pratensis* with places to feed, shelter, and overwinter, thereby increasing the number of *L. pratensis* migrating to cotton fields. This has led to a significant increase in *L. pratensis* populations in cotton fields, which has been emerging as one of the primary pests in cotton fields and exhibits a trend of further expansion [16,17,18].

In Xinjiang, *L. pratensis* has four generations per year and is characterized by a long life cycle, strong adaptability, high reproductive potential, and robust dispersal capabilities through flight [5,19,20]. Currently, due to the lack of more effective selective insecticides specifically targeting *L. pratensis* [21], chemical control relying on broad-spectrum insecticides remains the predominant method for managing *L. pratensis* in cotton fields [22,23]. This reliance has contributed to the gradual development of resistance in *L. pratensis* [24]. For instance, field populations of *L. pratensis* in seven semi-arid ecological transition zones between agricultural areas and grassland pastoral areas in Northern China show an increasing trend in resistance to high-efficacy cyfluthrin [8]. More critically, the unsustainable nature of broad-spectrum insecticides, along with their detrimental effects on non-target arthropods and human health, has become a widespread phenomenon and has increasingly raised concerns [25,26,27,28]. Research has found that acetamiprid, flonicamid thiamethoxam exhibit higher toxicity toward the cotton *Hippodamia variegata* (Coleoptera: Coccinellidae) than toward *Aphis gossypii* (Homoptera: Aphididae), demonstrating negative selectivity [29]. Low doses of acetamiprid and afidopyropen hurt the parasitic activity of *Lysiphlebia japonica* (Hymenoptera: Aphidiidae) [30]. Given these circumstances and the negative impacts of chemical control, there is an urgent need to develop practical and sustainable methods to replace the chemical control of *L. pratensis*. The use of natural enemies in pest control offers an approach to mitigating the damage caused by *L. pratensis* [31,32]. One study found that in agricultural ecosystems, natural enemies account for over 50% of pest control effectiveness [33]. The protection and utilization of natural enemies are fundamental to effectively implementing ecological control of *L. pratensis*, and assessing the extent of the control effect exerted by predatory natural enemies is a prerequisite for fully harnessing their potential in pest management [34]. For instance, Zhang et al. [35] surveyed the species of predatory natural enemies of *Tuta absoluta* (Lepidoptera: Gelechiidae) and found that Arachnida natural enemies accounted for more than half of all natural enemies, thereby providing a natural resource basis for the use of local natural enemies to control *T. absoluta*. Wang et al. [36] found that spiders from Thomisidae, Lycosidae, and Theridiidae exhibit relatively high predation of the nymphs of *Erythroneura apicalis* (Hemiptera: Cicadellidae), providing a scientific basis for the natural control of *E. apicalis*.

Numerous methods have been developed to evaluate the predatory ability of natural enemies. Zhang et al. [37] investigated the functional response and control capability of *Orius sauteri* (Hemiptera: Anthocoridae) against *Dendrothrips minowai* (Thysanoptera: Thripidae) using a predation functional response model. Since some radioactive isotopes can be transferred through the plant-herbivorous insect-predatory natural enemy food chain, the isotope detection rate of predatory natural enemies can track the feeding relationship between natural enemies and their prey [38]. Stam et al. [39] utilized radioactive isotopes (^32^P) to identify the types of natural enemies of *Nezara viridula* (Hemiptera: Pentatomidae) in soybeans. However, this was possible due to the strong flight dispersal ability of *L. pratensis* and the often brief duration of predator–*L. pratensis* interactions in the field [40]. Observing and recording the predatory habits and patterns of natural enemies on *L. pratensis* in the field is insufficient for analyzing the predation capabilities of a large number of predators. Furthermore, the use of radioactive isotope labelling is restricted by its complexity and associated risks [41], rendering it challenging to assess the role of predators in controlling *L. pratensis* [42,43]. However, molecular techniques for identifying the intestinal contents of predatory natural enemies have offered new avenues for elucidating the predation relationships between natural enemies and *L. pratensis* [44,45].

In this study, we designed species-specific PCR detection primers for *L. pratensis* and established a detection system to identify *L. pratensis* DNA in the intestinal contents of predatory natural enemies. Meanwhile, they were integrated with an indoor predation functional response model. Thereby, we evaluated of the predatory capacity of different natural enemies towards *L. pratensis*, systematically elucidated their control effects on *L. pratensis*, and provided a scientific basis for the innovative development of biological control technologies for *L. pratensis* in Xinjiang cotton fields.

## 2. Materials and Methods

### 2.1. Field Site and Sample Collection

From June to September 2024, during the peak season for *L. pratensis* infestations, sample collection was conducted at the Experimental Base of the Agricultural Science Research Institute of the First Division of the Xinjiang Production and Construction Corps (81°23′25″ E, 40°32′8″ N). The cotton in the experimental field was planted using the ‘one film, four rows’ planting pattern, with a plant spacing of 10 cm, a row spacing of (66 + 10) cm, and a planting density ranging from 150,000 to 180,000 plants per hectare. No pesticides were applied throughout the entire growth period. To collect *L. pratensis* and flying predatory natural enemies, the sweep net method was employed [46]. This method involved using an insect net with a mouth diameter of 38 cm, a net depth of 60 cm, and a pole length of 120 cm. During sweeping, the insect net was positioned at the middle layer of the plants, with each sweep covering 180°. Samples were collected once every 15 days. At each sampling, five sampling points were selected, and each sampling point was continuously swept with a net 100 times, with a minimum distance of 50 m between sampling points. Additionally, the GZY-XCQ type insect aspirator (Hubei Guangzhiying Technology Co., Ltd., Guangshui, Hubei, China) was used to collect ground-dwelling predatory natural enemies. For aspirator sampling, five additional sampling points were selected, each covering an area of 4 m^2^, collecting all visible arthropods until none remained at each sampling point, with a minimum distance of 50 m between sampling points [47]. Ground-dwelling arthropods were collected once every 15 days. A single specimen was placed in a 1.5 mL EP tube containing 100% alcohol (Sangon Bioengineering Co., Shanghai, China), labelled with a pencil, then stored at −20 °C for a minimum of 24 h upon return to the laboratory to prevent cross-contamination [48,49]. This procedure facilitated subsequent species identification and predation detection using molecular biology techniques.

### 2.2. Identification of Predatory Natural Enemies

First, the collected predatory natural enemies were identified at the species level based on morphological characteristics. Then, one sample was randomly selected from each species for further molecular identification. According to the manufacturer’s instructions, total DNA was extracted using the DNeasy Blood & Tissue Kit (QIAGEN Inc., Chatsworth, CA, USA). LCO-1490 and HCO-2198 primers [50] were employed to amplify the COI gene of the predatory natural enemies. The PCR reaction mixture was composed of 1 µL of genomic DNA, 9.5 µL of ddH_2_O (Sangon Bioengineering Co., Shanghai, China), 1 µL of each primer, and 12.5 µL of 2 × Taq PCR Master Mix (Sangon Bioengineering Co., Shanghai, China), resulting in a final volume of 25 µL. The PCR reaction procedure for amplifying the COI gene of predatory natural enemies using primers LCO-1490 and HCO-2198 is as follows: initial denaturation at 94 °C for 5 min, followed by 40 cycles at 94 °C for 30 s, primer annealing at 48 °C for 30 s, primer extension at 72 °C for 46 s, and a final extension at 72 °C for 10 min. The amplification reaction was conducted using a C1000™ Thermal Cycler (Bio-Rad Laboratories Inc., Hercules, CA, USA). The PCR product was visualized on a 1% agarose gel containing ethidium bromide (Sangon Bioengineering Co., Shanghai, China) (0.5 µg/mL) under UV light. A successfully amplified PCR product was submitted to Sangon Bioengineering (Shanghai) Co., Ltd. for sequencing. The generated sequences were analyzed alongside relevant predator COI gene sequences obtained from GenBank and recent publications (Table 1). The alignment based on COI sequence data was performed using MAFFT v.6 and manually edited with BioEdit v.7.2.3. Phylogenetic analyses were conducted using IQ-TREE v.1.6.12 for maximum likelihood (ML). Confidence levels for the nodes were determined through 1000 bootstrap replicates. Phylogenetic trees were plotted in FigTree v.1.4.3 and annotated in iTOL v.6 (https://itol.embl.de/, accessed on 28 December 2024). The sequences obtained from the sequencing were submitted to GenBank (Table 2).

### 2.3. Analysis of Molecular Intestinal Contents

The fundamental principles and methodologies of the molecular intestinal contents analysis were founded upon previously published studies concerning the relationship between insects and their predatory natural enemies [70,71,72,73]. Total DNA was extracted from *L. pratensis* using the method described in Section 2.2, and the COI gene was amplified. The successful PCR products were submitted to Sangon Bioengineering (Shanghai) Co., Ltd. for sequencing, and the resulting sequences have been deposited in GenBank (PV875978). Specific primers targeting *L. pratensis* were designed to detect the species of predatory natural enemies of *L. pratensis* present in the cotton field ecosystem. The design of these specific primers adhered to the commonly employed protocol for molecular gut content analysis [74]. Initially, specific primer pairs for *L. pratensis* were developed using the sequences PV875978 in conjunction with the NCBI Primer-BLAST tool (https://www.ncbi.nlm.nih.gov/tools/primer-blast/, accessed on 10 June 2024), and their specificity was verified against the arthropod database. The COI gene sequence of *L. pratensis* (PV875978) was aligned with the complete COI regions of other Miridae species available in GenBank using MAFFT v.6 [75], and the specificity of the primer pairs was preliminarily assessed [76]. Following testing with Primer-BLAST, one primer pair was selected (forward: MC-3F: TTGGTGCGCCAGATATAGCA, Tm = 56.6 °C; reverse: MC-3R: CGGTGATTCCCACCGATCAT, Tm = 57.6 °C), producing an amplification product of 315 bp. The PCR reagents used for optimizing the temperature settings were the same as those described in Section 2.2. The optimal PCR thermal cycling protocol consisted of an initial denaturation at 94 °C for 5 min, followed by 40 cycles of 94 °C for 30 s, 57 °C for 30 s, and 72 °C for 46 s, concluding with a final extension of 10 min at 72 °C. In the laboratory, the DNA of *L. pratensis* was used as a positive control, and the DNA of other pest samples collected in the same cotton field in the same living environment as *L. pratensis* was used as a negative control to test the specificity of the primers (Table 3).

We extracted the total DNA from all collected predatory natural enemies using the method described in Section 2.2. PCR amplification and electrophoretic detection were conducted using specific primers for *L. pratensis* (MC-3F, MC-3R). The presence or absence of the target fragment was used to determine the outcome. Predator individuals demonstrating clear target fragments in the electrophoresis analysis were classified as positive reaction individuals and thus identified as predatory natural enemies of *L. pratensis*. Conversely, predator individuals lacking the target fragments in the electrophoresis analysis were categorized as negative reaction individuals and consequently determined not to be natural enemies of *L. pratensis*.

### 2.4. Predatory Ability of Natural Enemies on L. pratensis

Of the 23 species of natural enemies tested, four tested positive, all spiders. The four species of spiders that tested positive were further studied to determine their predatory ability against *L. pratensis*. Both *L. pratensis* and the spiders were collected from cotton fields (81°7′36″ E, 40°38′17″ N) in Alar City, Xinjiang. *Lygus pratensis* were maintained in an artificial climate chamber (Ningbo Jiangnan Instrument Factory, Ningbo, China) at the Green Pest Control Laboratory of Tarim University using insect rearing nets measuring 35 cm × 35 cm × 35 cm and fed fresh cauliflower daily under conditions of 25 ± 0.5 °C with a relative humidity of 70 ± 10% and a light/dark cycle of 14 h/10 h, facilitating the stable reproduction of *L. pratensis* [77]. Adult natural enemies collected from the field were housed in transparent plastic culture bottles (80 mm × 90 mm, diameter × height). The environmental conditions were the same as those of *L. pratensis*, with only one spider per bottle. Each bottle contained a fresh cotton leaf, and the petiole was wrapped in a wet cotton ball to maintain freshness for an extended period. Following a 24 h starvation period, a fresh cotton leaf of uniform size (with the petiole wrapped in a wet, lint-free cotton ball) was placed in each culture bottle. Test *L. pratensis* fourth to fifth instar nymphs and adults were introduced into the bottles [78], with six density treatments established: 5, 10, 15, 20, 25, and 30 individuals per bottle. These were then placed with a natural enemy that had been starved for 24 h. The bottles were placed in the artificial climate chamber under the same conditions. After 24 h, the number of surviving *L. pratensis* in each culture bottle was recorded, with four replicates conducted for each density treatment.

The data were analyzed to evaluate the functional response of four species of spiders to different *L. pratensis* stage. Functional response data analysis included two steps. First, logistic regression was used to determine the type of functional response [79,80]. Specifically, the following polynomial function (1) was used to examine the relationship between the number of prey consumed (*N*a) and the initial density (*N*) of the prey:

*N*a/*N* = a + b*N* + c*N*^2^ + d*N*^3^ (1)

where *N*a is the number of *L. pratensis* consumed; *N* is the initial density of *L. pratensis*; a, b, c, and d are parameters; and parameter estimation was performed using the least squares method. When the coefficient b < 0, the functional response is type II; when b > 0, the functional response is type III [79,80].

Second, the ‘disc equation’ was used to obtain estimates for handling time (*Th*) and attack rate (*a′*) [79,81]. Equations (2) and (3) represent Holling’s Type II and Type III functional response models, respectively, as given below:

*N*a = *a′TN*/(1 + *a′ThN*) (2)

*N*a = *a′TN*^2^/(1 + *a′ThN*^2^) (3)

In this formula, “*N*” represents the initial density of prey; “*N*a” is the number of prey encountered per predator; “*a′*” denotes the instantaneous attack rate; “*T*” represents the time that predator and prey are exposed to each other (1 d); and “*Th*” denotes the “handling time” associated with each prey eaten.

According to the estimated attack rate and handling time, the search efficiency is calculated [82], as follows:

*S* = *a′*/(1 + *a′ThN*) (4)

where “*S*” is the search efficiency, and the other parameter denotations are the same as those in Formulas (1) and (2). The daily maximum predation rate (*T/Th*) and theoretical predation (*a′/Th*) were also calculated.

### 2.5. Data Analysis

The functional response types were estimated using logistic regression in GraphPad Prism 10.4.2 software. Statistical analyses of all experimental data were performed using SPSS 25.0 (IBM SPSS Inc., Chicago, IL, USA), while Origin 2021 (OriginLab Corporation, Northampton, MA, USA) was used for figure generation.

## 3. Results

### 3.1. Identification of Predatory Natural Enemies in Cotton Fields

**Figure 1:**
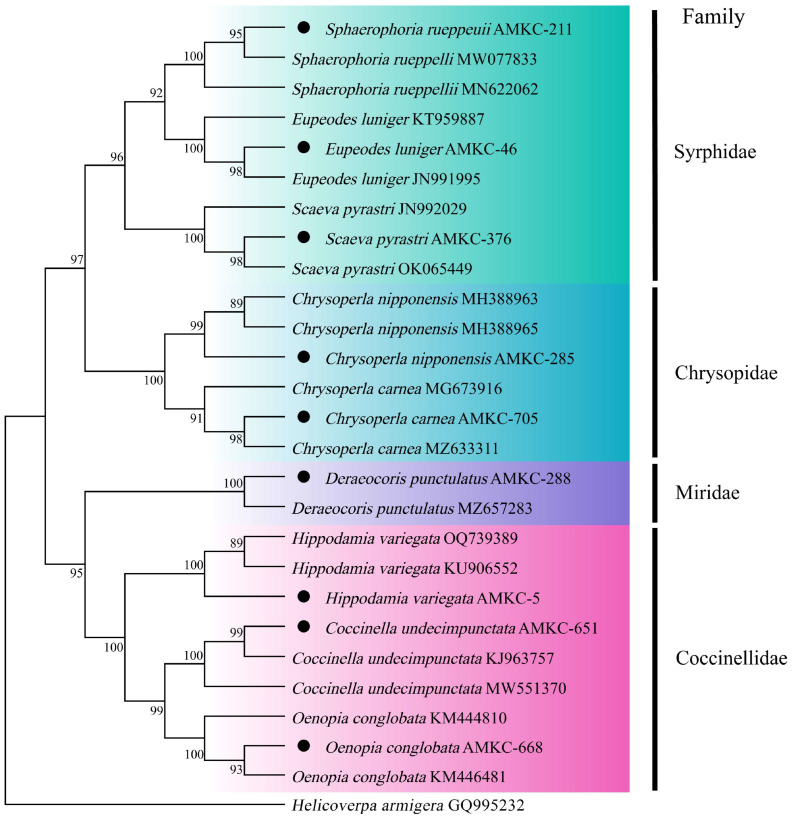
Phylogenetic tree of predatory natural enemies of insects in the cotton field based on the COI gene. The black dots indicate that sequencing samples from predatory natural enemies contained this gene sequence.

A total of 826 predatory natural enemies were collected from cotton fields. These predatory natural enemies were identified as 23 species based on morphological characteristics. The sequences resulting from sequencing were compared with the most similar gene sequences available in the GenBank database and those reported in the literature (Table 1). A phylogenetic tree was constructed using the maximum likelihood (ML) method. Among these, nine insect samples from the Insecta were analyzed. The COI gene sequence of *Helicoverpa armigera* (Lepidoptera: Noctuidae) (GQ995232) was used as an outgroup for constructing the ML phylogenetic tree of the insecta predatory natural enemy community in cotton fields. The clustering results indicated that the outgroup *H. armigera* and the nine predatory natural enemy samples formed sister groups, with no overlap in species identified after clustering. Each of the nine predatory natural enemy species constituted a monophyletic group and was distinguishable. Various predatory natural enemy species were distinctly classified into four orders, four families, and nine species (Figure 1).

**Figure 2:**
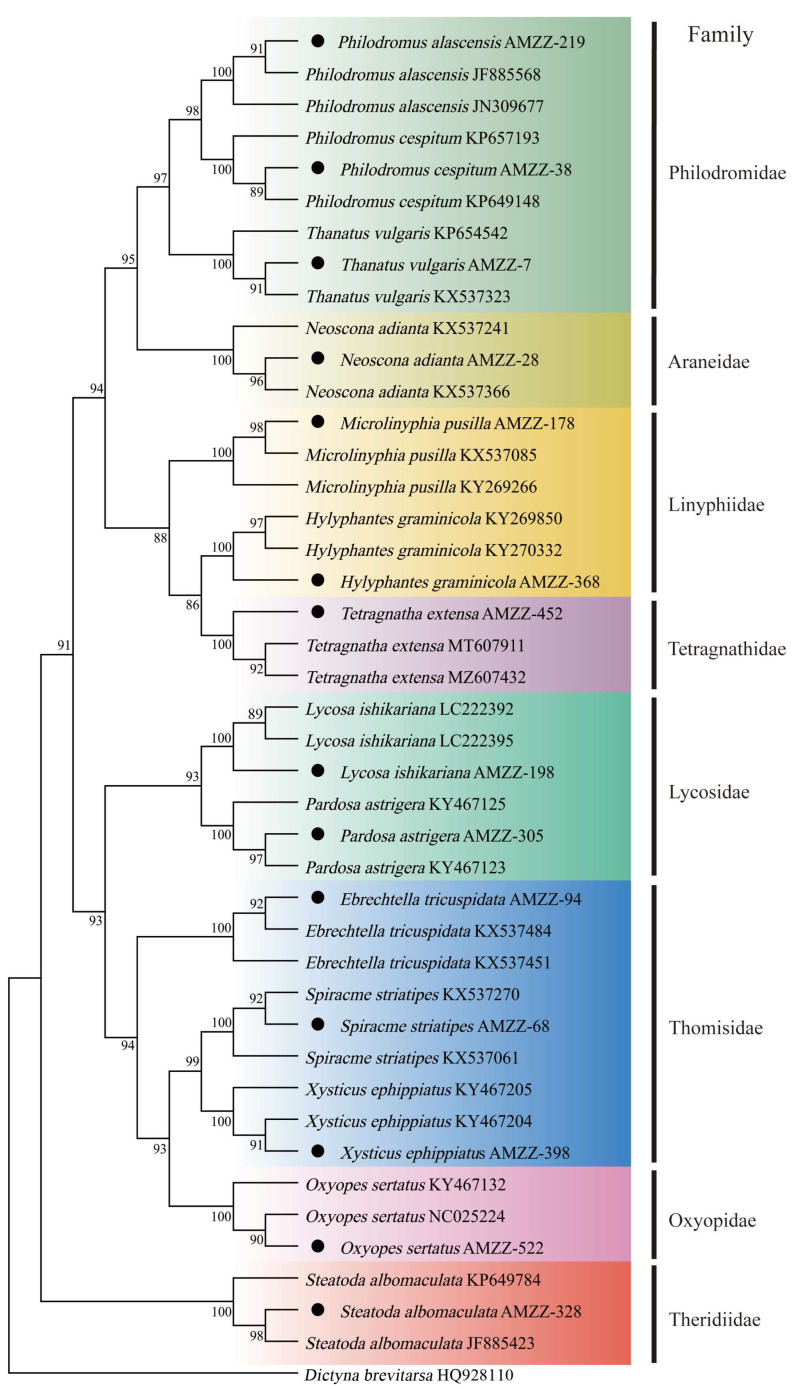
Phylogenetic tree of predatory natural enemies of spiders in the cotton field based on the COI gene. The meaning of the black dots is the same as above.

A total of fourteen spider samples from the Arachnida were analyzed. The COI gene sequence of *Dictyna brevitarsa* (Araneae: Dictynidae) (HQ928110) was utilized as an outgroup to construct a maximum likelihood (ML) phylogenetic tree for the predatory natural enemy community within the Arachnida in cotton fields. The clustering results indicated that the outgroup *D. brevitarsa* and the fourteen spider samples were classified as sister groups. Following clustering, the fourteen spider samples exhibited no species overlap, with each of the 14 spider species forming a monophyletic group. This distinguished different spiders into one order, eight families, and fourteen species (Figure 2).

**Figure 3:**
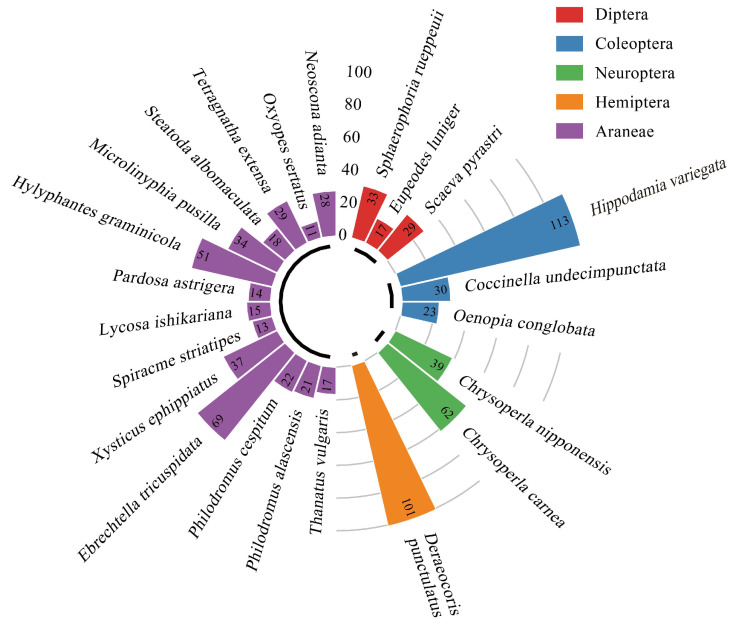
Types and numbers of predatory natural enemies in cotton fields.

Consequently, the predatory natural enemies present in cotton fields are classified into two classes, five orders, and twelve families. Among these, there are 9 species of Insecta, comprising a total of 447 individuals, which account for 54.12% of the overall population of predatory natural enemies. Additionally, there are 14 species of Arachnida, amounting to 379 individuals, representing 45.88% of the total. Within the 4 orders of Insecta, *Hippodamia variegata* (Coleoptera: Coccinellidae) exhibits the highest population, featuring 113 individuals, which constitutes 13.68% of the total number of predatory natural enemies. This is followed by *Deraeocoris punctulatus* (Hemiptera: Miridae), with 101 individuals accounting for 12.23%. Among Arachnida, *Ebrechtella tricuspidata* (Araneae: Thomisidae) has the highest population, with 69 individuals, corresponding to 8.35%, followed by *Hylyphantes graminicola* (Araneae: Araneae), which consists of 51 individuals, amounting to 6.17% (Figure 3).

### 3.2. Determination of Predatory Natural Enemies of L. pratensis

**Figure 4:**
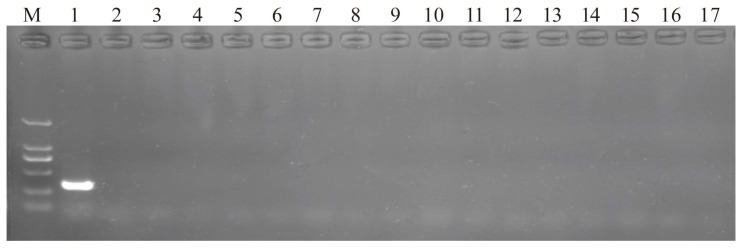
PCR product electrophoresis diagram for primer specificity verification: (M) DNA marker-DL2000; (1) *L. pratensis*; (2) *A. lucorum*; (3) *A. lineoatus*; (4) *E. maracandica*; (5) *C. viridis*; (6) *T. punctipennis*; (7) *C. pipiens*; (8) *F. canicularis*; (9) *A. gossypii*; (10) *A. gossypii*; (11) *A. craccivora*; (12) *T. vaporariorum*; (13) *H. armigera*; (14) *S. exigua*; (15) *A. segetum*; (16) *T. tabaci*; (17) *T. dunhuangensis*.

Primer specificity testing results indicated no non-target amplification products (Figure 4). PCR amplification was conducted using *L. pratensis*-specific primers with DNA samples from predatory natural enemies collected from cotton fields to ascertain the positive detection rate among the various samples. Since DNA extraction was unsuccessful for ten natural enemies, a total of 806 natural enemies were tested. The findings revealed that out of 806 natural enemies evaluated, 5.58% of the predators tested positive for *L. pratensis* (Table 4). All positive results were from predatory natural enemies within Araneae, specifically *E. tricuspidata*, *X. ephippiatus*, *H. graminicola*, and *O. sertatus*. Notably, *E. tricuspidata* exhibited the highest detection rate, reaching 42.42% (Table 4).

### 3.3. The Predatory Effects of Four Species of Spiders on L. pratensis

#### 3.3.1. The Predatory Function of Four Species of Spiders on *L. pratensis*

**Figure 5:**
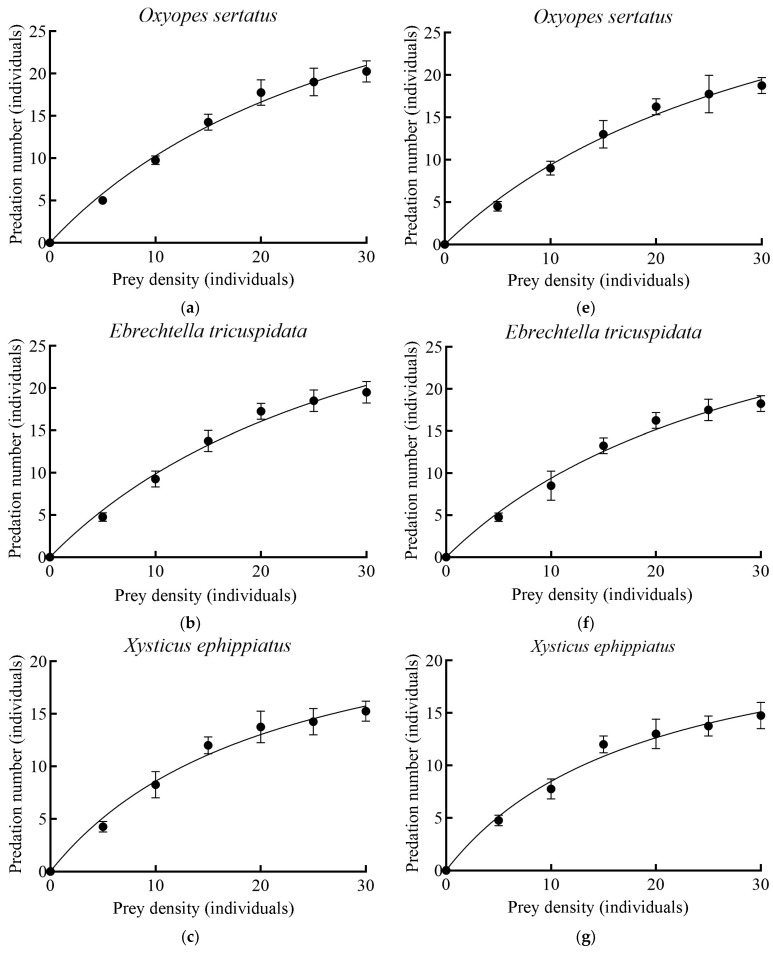

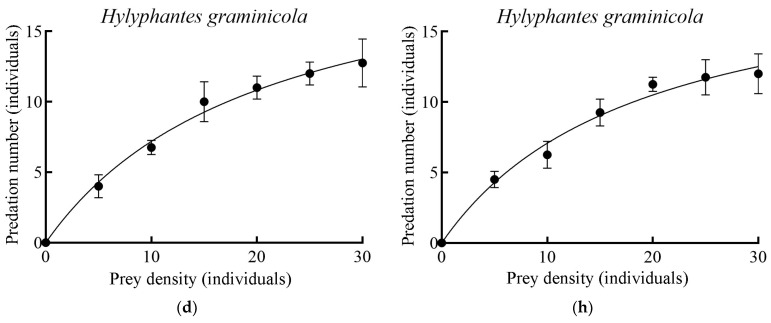
The predation number for the four spider species on the fourth to fifth instar nymphs and adults of *L. pratensi*: (**a**–**d**) Four spider species’ functional response models to fourth to fifth instar nymphs of *L. pratensis*; (**e**–**h**) Four spider species’ functional response models to adults of *L. pratensis*. Error bars represent the standard error for each set of data.

The functional responses of four spider species towards fourth to fifth instar nymphs and adults of *L. pratensis* conform to the Holling II model (Table 5). In this model, predation rates increase with rising prey density; however, the increase in predation growth rate diminishes as prey density increases. The predation number observed for the four spider species on the fourth to fifth instar nymphs and adults (1 d) of *L. pratensis* was as follows: *O. sertatus* > *E. tricuspidata* > *X. ephippiatus* > *H. graminicola* (Figure 5).

The results of the predation functional response parameters for four spider species targeting fourth to fifth instar nymphs and adults of *L. pratensis* indicated that the theoretical predation (*a′*/*Th*) and daily maximum predation rate (*T*/*Th*) adhered to the order *O. sertatus* > *E. tricuspidata* > *X. ephippiatus* > *H. graminicola*, which is consistent with the results presented in Figure 5. When comparing fourth to fifth instar nymphs with adults; the theoretical predation (*a′*/*Th*) and daily maximum predation rate (*T*/*Th*) of the four spider species were found to be higher for fourth to fifth instar nymphs than for adults. Among these species, *O. sertatus* exhibited the highest theoretical predation (*a′*/*Th*), achieving 60.86 for fourth to fifth instar nymphs and 50.25 for adults. In contrast, *H. graminicola* displayed the lowest theoretical predation (*a′*/*Th*), recording 23.71 against fourth to fifth instar nymphs and 22.14 against adults. Furthermore, *O. sertatus* possessed the highest daily maximum predation rate (*T*/*Th*) at 45.45 for fourth to fifth instar nymphs and 41.66 for adults, while *H. graminicola* had the lowest daily maximum predation rate (*T*/*Th*) at 22.22 for fourth to fifth instar nymphs and 20.41 for adults. The instantaneous attack rate (*a′*) of *O. sertatus* on fourth to fifth instar nymphs was highest at 1.339, whereas the instantaneous attack rate (*a′*) of *X. ephippiatus* on adults reached its peak at 1.290. Additionally, the handling time (*Th*) recorded for *O. sertatus* against fourth to fifth nymphs and adults was 0.022 days per individual and 0.024 days per individual, respectively (Table 6).

#### 3.3.2. Searching Effects of Four Species of Spiders on *L. pratensis*

**Figure 6:**
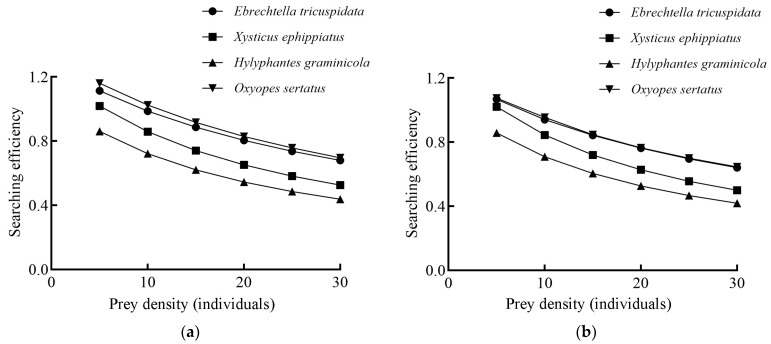
Searching effects of four species of spiders on *L. pratensis*: (**a**) Searching effects of four species of spiders on fourth to fifth instar nymphs; (**b**) Searching effects of four species of spiders on adults.

The search effects of four spider species on the fourth to fifth instar nymphs and adults of *L. pratensis* were negatively correlated with prey density. The search effects were ranked as follows: *O. sertatus* > *E. tricuspidata* > *X. ephippiatus* > *H. graminicola* (Figure 6). When the prey density was set at five individuals per bottle, *O. sertatus* demonstrated the highest search effect, with values of 1.16 for fourth to fifth nymphs and 1.074 for adults. In contrast, *H. graminicola* exhibited the lowest search effect, recording values of 0.86 for fourth to fifth nymphs and 0.857 for adults.

## 4. Discussion

The results of our study indicate that the predatory natural enemies of *L. pratensis* in cotton fields are *O. sertatus*, *E. tricuspidata*, *X. ephippiatus*, and *H. graminicola*. Currently, there are no studies indicating that these four species of spiders can prey on *L. pratensis*. Previous research has also examined the natural enemies of Miridae; for instance, Tong et al. [83] reported that, in experiments, *E. tricuspidata* can prey upon both nymphs and adults of *Apolygus lucorum* (Hemiptera: Miridae) and *Lygus lineolaris* (Hemiptera: Miridae). It can be inferred that *E. tricuspidata* may also target *L. pratensis*. Our study successfully detected *L. pratensis* DNA in *E. tricuspidata*, providing further evidence of this species’ predation on *L. pratensis*. *Oxyopes sertatus*, *X. ephippiatus*, and *H. graminicola* were also observed to prey on *L. pratensis*. Although four spider species were identified as capable of preying on *L. pratensis*, our study still has certain limitations; for example, the detection rate for *L. pratensis* is relatively low. As target pests vary in size depending on their life stage, smaller target insects have less DNA content. They are more easily digested and degraded after being consumed by natural enemies. Additionally, larger predatory natural enemies have stronger digestive dilution effects in their intestines, so when consuming the same target pests, the target DNA is more easily diluted and degraded [34,84]. At the same time, *L. pratensis* has strong flight dispersal capabilities and is not easily captured by natural enemies [40], which significantly influenced the accuracy of prey DNA detection rates. Further research is warranted in this area to enhance the understanding of the diversity of natural enemies of *L. pratensis*.

We conducted indoor experiments to evaluate the predation response of four spider species toward fourth to fifth instar nymphs and adult *L. pratensis*. The results indicated that the theoretical predation (*a′*/*Th*), daily maximum predation rate (*T*/*Th*), and search effects of the four spider species on the fourth to fifth instar nymphs and adults of *L. pratensis* followed the order *O. sertatus* > *E. tricuspidata* > *X. ephippiatus* > *H. graminicola*. Quan et al. [85] studied the predatory effects of *E. tricuspidata*, *Pardosa astrigera* (Araneae: Lycosidae), *O. sertatus*, and *Dolomedes sulfureus* (Araneae: Pisauridae) on *Corythucha ciliata* (Hemiptera: Tingidae) and found that the functional responses of *E. tricuspidata* and *O. sertatus* to *C. ciliata* also conformed to the Holling II model, which is consistent with the findings of our study. Spiders are highly sensitive to large-scale landscape factors, and non-crop habitats in agricultural landscapes are important sites for spider migration, dispersal, and reproduction [86]. Therefore, avoiding monoculture crop planting and protecting the landscape structure of non-crop habitats can significantly increase spider diversity and population density, thereby promoting spiders’ ecological regulatory role [87]. This research comprehensively evaluated the functional responses of four spider species towards *L. pratensis* through predation functions and search effects, thereby providing theoretical support for spider-based *L. pratensis* control in agricultural fields. By utilizing these natural enemies, it is hoped that dependence on chemical pesticides can be reduced, promoting green pest control and ecological safety in cotton production and driving the development of sustainable agriculture.

Recent studies have demonstrated that a significant increase in the population of the same or multiple species of predatory natural enemies within a particular spatial range can lead to localized dispersion. This phenomenon causes interference among predators, subsequently reducing the efficiency of natural enemies in locating their prey [88]. Such interactions may result in intraguild predation (IGP) and lethal interference competition [89,90,91]. When multiple prey species coexist, the selective targeting of different prey by the same predator species can also adversely affect their predation capacity. In addition, prey of different ages also affect the predatory ability of natural enemies due to differences in dimensions and mobility [92,93]. This study only examined the predatory capabilities of four spider species on fourth to fifth instar nymphs and adult *L. pratensis*. Future research should focus on the predatory ability of natural enemies across all life stages of *L. pratensis*, the natural enemy *L. pratensis* food web structure, and the environmental factors that affect the efficacy of natural enemy control, providing a foundational basis for in-depth exploration of the predation mechanisms of these natural enemies in cotton field systems and identifying pathways to enhance their natural regulation of *L. pratensis* within cotton field ecosystems.

## 5. Conclusions

This study utilized a combined technical approach involving molecular biology detection and predation function analysis to elucidate the predatory natural enemies of the cotton field *L. pratensis*. The identified natural enemies include *O. sertatus*, *E. tricuspidata*, *X. ephippiatus*, and *H. graminicola*. The predatory responses of these natural enemies towards the fourth to fifth instar nymphs and adults of the cotton field *L. pratensis* conform to the Holling II model, with *O. sertatus* and *E. tricuspidata* exhibiting particularly promising control potential. These findings provide reliable scientific evidence for assessing the control potential of natural enemies against *L. pratensis*. Consequently, using natural enemies for the biological control of cotton field *L. pratensis* shows promise as a sustainable biological control strategy.

## Figures and Tables

**Table 1:** ALAST results and reference sequence sources of gene sequences in GenBank.

Natural Enemy Species			Genbank Accession Number	Homology (%)	References
Order	Family	Species *			
Diptera	Syrphidae	*Sphaerophoria rueppeuii* Wiedemann	MN622062	99.85	[51]
			MW077833	99.70	[52]
		*Eupeodes luniger* Meigen	JN991995	99.70	[53]
			KT959887	99.54	[54]
		*Scaeva pyrastri* Linnaeus	JN992029	99.85	[53]
			OK065449	99.85	[55]
Coleoptera	Coccinellidae	*Hippodamia variegata* Goeze	OQ739389	100	[56]
			KU906552	99.85	[57]
		*Coccinella undecimpunctata* Linnaeus	KJ963757	99.70	[58]
			MW551370	99.54	[59]
		*Oenopia conglobata* Linnaeus	KM446481	99.85	[60]
			KM444810	99.39	[60]
Neuroptera	Chrysopidae	*Chrysoperla nipponensis* Okamoto	MH388963	99.84	[61]
			MH388965	99.84	[61]
		*Chrysoperla carnea* Stephens	MG673916	99.30	[62]
			MZ633311	99.30	[55]
Hemiptera	Miridae	*Deraeocoris punctulatus* Fallen	MZ657283	99.85	[55]
Araneae	Philodromidae	*Thanatus vulgaris* Simon	KP654542	100	[63]
			KX537323	99.85	[64]
		*Philodromus alascensis* Keyserling	JN309677	97.57	[63]
			JF885568	96.66	[63]
		*Philodromus cespitum* Walckenaer	KP657193	99.93	[63]
			KP649148	99.93	[63]
	Thomisidae	*Ebrechtella tricuspidata* Fabricius	KX537484	100	[64]
			KX537451	100	[64]
		*Xysticus ephippiatus* Simon	KY467204	99.85	[65]
			KY467205	99.39	[65]
		*Spiracme striatipes* L.Koch	KX537061	100	[64]
			KX537270	99.85	[64]
	Lycosidae	*Lycosa ishikariana* Saito	LC222392	99.69	[66]
			LC222395	99.53	[66]
		*Pardosa astrigera* L. Koch	KY467123	99.54	[65]
			KY467125	98.63	[65]
	Linyphiidae	*Hylyphantes graminicola* Sundevall	KY270332	99.68	[64]
			KY269850	99.68	[64]
		*Microlinyphia pusilla* Sundevall	KX537085	99.85	[64]
			KY269266	99.85	[64]
	Theridiidae	*Steatoda albomaculata* De Geer	JF885423	99.85	[63]
			KP649784	99.70	[63]
	Tetragnathidae	*Tetragnatha extensa* Linnaeus	MZ607432	98.33	[55]
			MT607911	98.33	[67]
	Oxyopidae	*Oxyopes sertatus* L. Koch	KY467132	100	[65]
			NC025224	100	[68]
	Araneidae	*Neoscona adianta* Walckenaer	KX537366	99.54	[64]
			KX537241	99.54	[64]
Araneae	Dictynidae	*Dictyna brevitarsa* Emerton	HQ928110		[63]
Lepidoptera	Noctuidae	*Helicoverpa armigera* Hübner	GQ995232		[69]

* Two reference sequences were selected for each species, but only one reference sequence was available for *D. punctulatus* in the literature. *D. brevitarsa* (HQ928110) and *H. armigera* (GQ995232) sequences were included as an outgroup in all analyses. No sequence alignment was performed.

**Table 2:** Sample sequence numbers and GenBank accession numbers.

Natural Enemy Species			Sample Number	GenBank Accession Number
Order	Family	Species		
Diptera	Syrphidae	*Sphaerophoria rueppeuii* Wiedemann	AMKC-211	PV875959
		*Eupeodes luniger* Meigen	AMKC-46	PV875960
		*Scaeva pyrastri* Linnaeus	AMKC-376	PV875961
Coleoptera	Coccinellidae	*Hippodamia variegata* Goeze	AMKC-5	PV875964
		*Coccinella undecimpunctata* Linnaeus	AMKC-651	PV875965
		*Oenopia conglobata* Linnaeus	AMKC-668	PV875966
Neuroptera	Chrysopidae	*Chrysoperla nipponensis* Okamoto	AMKC-285	PV875962
		*Chrysoperla carnea* Stephens	AMKC-705	PV875963
Hemiptera	Miridae	*Deraeocoris punctulatus* Fallen	AMKC-288	PV875967
Araneae	Philodromidae	*Thanatus vulgaris* Simon	AMZZ-7	PV875979
		*Philodromus alascensis* Keyserling	AMZZ-219	PV875968
		*Philodromus cespitum* Walckenaer	AMZZ-38	PV875969
	Thomisidae	*Ebrechtella tricuspidata* Fabricius	AMZZ-94	PV875970
		*Xysticus ephippiatus* Simon	AMZZ-398	PV875971
		*Spiracme striatipes* L.Koch	AMZZ-68	PV875981
	Lycosidae	*Lycosa ishikariana* Saito	AMZZ-198	PV875982
		*Pardosa astrigera* L. Koch	AMZZ-305	PV875973
	Linyphiidae	*Hylyphantes graminicola* Sundevall	AMZZ-368	PV875974
		*Microlinyphia pusilla* Sundevall	AMZZ-178	PV875975
	Theridiidae	*Steatoda albomaculata* De Geer	AMZZ-328	PV875976
	Tetragnathidae	*Tetragnatha extensa* Linnaeus	AMZZ-452	PV875980
	Oxyopidae	*Oxyopes sertatus* L. Koch	AMZZ-522	PV875972
	Araneidae	*Neoscona adianta* Walckenaer	AMZZ-28	PV875977

**Table 3:** Types of cotton field pests used for specific detection.

Order	Family	Species
Hemiptera	Pentatomidae	*Eurydema maracandica* Oshanin
	Cicadellidae	*Cicadella viridis* Linnaeus
	Miridae	*Apolygus lucorum* Meyer-Dür
		*Adelphocoris lineoatus* Goeze
Homoptera	Aleyrodidae	*Trialeurodes vaporariorum* Westwood
	Aphididae	*Aphis gossypii* Glover
		*Acyrthosiphon gossypii* Mordvilko
		*Aphis craccivora* Koch
Diptera	Chironomidae	*Tanypus punctipennis* Meigen
	Culicidae	*Culex pipiens* Linnaeus
	Muscidae	*Fannia canicularis* Linnaeus
Lepidoptera	Noctuidae	*Helicoverpa armigera* Hübner
		*Spodoptera exigua* Hübner
		*Agrotis segetum* Denis et Schiffermüller
Thysanoptera	Thripidae	*Thrips tabaci* Lindeman
Acarina	Tetranychidae	*Tetranychus dunhuangensis* Wang

**Table 4:** Detection of predatory natural enemies of *L. pratensis* in cotton fields.

Natural Enemy Species			Number of Collected	Number of Detected	Number of Positive	Positive of DNA Detection (%) *
Order	Family	Species				
Diptera	Syrphidae	*Sphaerophoria rueppeuii* Wiedemann	33	33	0	0
		*Eupeodes luniger* Meigen	17	16	0	0
		*Scaeva pyrastri* Linnaeus	29	29	0	0
Coleoptera	Coccinellidae	*Hippodamia variegata* Goeze	113	109	0	0
		*Coccinella undecimpunctata* Linnaeus	30	28	0	0
		*Oenopia conglobata* Linnaeus	23	23	0	0
Neuroptera	Chrysopidae	*Chrysoperla nipponensis* Okamoto	39	38	0	0
		*Chrysoperla carnea* Stephens	62	62	0	0
Hemiptera	Miridae	*Deraeocoris punctulatus* Fallen	101	100	0	0
Araneae	Philodromidae	*Thanatus vulgaris* Simon	17	16	0	0
		*Philodromus alascensis* Keyserling	21	19	0	0
		*Philodromus cespitum* Walckenaer	22	19	0	0
	Thomisidae	*Ebrechtella tricuspidata* Fabricius	69	66	28	42.42
		*Xysticus ephippiatus* Simon	37	37	7	18.92
		*Spiracme striatipes* L.Koch	13	13	0	0
	Lycosidae	*Lycosa ishikariana* Saito	15	15	0	0
		*Pardosa astrigera* L. Koch	14	14	0	0
	Linyphiidae	*Hylyphantes graminicola* Sundevall	51	51	6	11.76
		*Microlinyphia pusilla* Sundevall	34	32	0	0
	Theridiidae	*Steatoda albomaculata* De Geer	18	18	0	0
	Tetragnathidae	*Tetragnatha extensa* Linnaeus	29	29	0	0
	Oxyopidae	*Oxyopes sertatus* L. Koch	11	11	4	36.37
	Araneidae	*Neoscona adianta* Walckenaer	28	28	0	0

* Positive DNA detection (%) = (number of positive individuals of natural enemies/number of detected natural enemies) ×100.

**Table 5:** Logistic regression results determining the type of functional response.

*L. pratensis* Stage	Natural Enemies	Parameters	*R* ^2^
Fourth to fifth instar nymphs	*E* *. tricuspidata*	*Na/N* = 0.961 − 0.008*N* + 0.0006*N*^2^ − 8.642 × 10^−7^*N*^3^	0.733
	*X* *. ephippiatus*	*Na/N* = 1.321 − 0.029*N* + 0.002*N*^2^ − 3.901 × 10^−5^*N*^3^	0.774
	*H* *. graminicola*	*Na/N* = 0.884-0.019*N* + 0.0002*N*^2^ − 3.211 × 10^−6^*N*^3^	0.727
	*O* *. sertatus*	*Na/N* = 1.018 − 0.012*N* + 0.001*N*^2^ − 9.631 × 10^−6^*N*^3^	0.853
Adults	*E* *. tricuspidata*	*Na/N* = 1.021 − 0.021*N* + 0.001*N*^2^ − 2.778 × 10^−5^*N*^3^	0.647
	*X* *. ephippiatus*	*Na/N* = 1.056 − 0.027*N* + 0.0005*N*^2^ − 8.642 × 10^−6^*N*^3^	0.821
	*H* *. graminicola*	*Na/N* = 1.292 − 0.105*N* + 0.005*N*^2^ − 8.877 × 10^−5^*N*^3^	0.831
	*O* *. sertatus*	*Na/N* = 0.991 − 0.016*N* + 0.001*N*^2^ − 1.272 × 10^−5^*N*^3^	0.661

**Table 6:** Functional response parameters of different natural enemies preying on *L. pratensis*.

*L. pratensis* Stage	Natural Enemies	Holling Equation	*R* ^2^	Instantaneous Attack Rate (*a′*)	Handling Time (*Th*)	Daily Maximum Predation Rate (*T*/*Th*)	Theoretical Predation (*a′*/*Th*)
Fourth to fifth instar nymphs	*E. tricuspidata*	*N*a = 1.276*N*/(1 + 0.029*N*)	0.975	1.276	0.023	43.48	55.48
	*X. ephippiatus*	*N*a = 1.256*N*/(1 + 0.046*N*)	0.961	1.256	0.037	27.03	33.95
	*H. graminicola*	*N*a = 1.067*N*/(1 + 0.048*N*)	0.955	1.067	0.045	22.22	23.71
	*O. sertatus*	*N*a = 1.339*N*/(1 + 0.029*N*)	0.976	1.339	0.022	45.45	60.86
Adults	*E. tricuspidata*	*N*a = 1.231*N*/(1 + 0.031*N*)	0.969	1.231	0.025	40.00	49.24
	*X. ephippiatus*	*N*a = 1.290*N*/(1 + 0.053*N*)	0.962	1.290	0.041	24.39	31.46
	*H. graminicola*	*N*a = 1.085*N*/(1 + 0.053*N*)	0.951	1.085	0.049	20.41	22.14
	*O. sertatus*	*N*a = 1.206*N*/(1 + 0.029*N*)	0.968	1.206	0.024	41.66	50.25

## Data Availability

The original contributions presented in this study are included in the article. Further inquiries can be directed to the corresponding author.

## References

[1] Fang L., Wang Q., Hu Y., Jia Y.H., Chen J.D., Liu B.L., Zhang Z.Y., Guan X.Y., Chen S.Q., Zhou B.L. (2017). Genomic analyses in cotton identify signatures of selection and loci associated with fiber quality and yield traits. Nat. Genet..

[2] Ridley W., Devadoss S. (2023). Competition and trade policy in the world cotton market: Implications for us cotton exports. Am. J. Agric. Econ..

[3] Hu H.Y., Ma Y.J., Shan Y.P., Song X.P., Wang D., Ren X.L., Li J., Niu Y.B., Wu C.C., Ma X.Y. (2023). Effects of adjuvants on physicochemical properties of nanopesticide applied by plant protection unmanned aerial vehicles and control of aphids in cotton field. Cotton Sci..

[4] Liu B., Li H.Q., Ali A., Li H.B., Liu J., Yang Y.Z., Lu Y.H. (2015). Effects of temperature and humidity on immature development of *Lygus pratensis* (L.) (Hemiptera: Miridae). J. Asia-Pac. Entomol..

[5] Zhang L.J., Cai W.Z., Luo J.Y., Zhang S., Wang C.Y., Lv L.M., Zhu X.Z., Wang L., Cui J.J. (2017). Phylogeographic patterns of *Lygus pratensis* (Hemiptera: Miridae): Evidence for weak genetic structure and recent expansion in Northwest China. PLoS ONE.

[6] Zhang R.F., Wang W., Liu H.Y., Yao J. (2022). Host plants species and seasonal succession host feeding of *Lygus pratensis* (Heteroptera: Miridae). Xinjiang Agric. Sci..

[7] Fitt G.P., Mares C.L., Llewellyn D.J. (1994). Field evaluation and potential ecological impact of transgenic cottons (*Gossypium hirsutum*) in Australia. Biocontrol Sci. Technol..

[8] Tan Y., Ma Y., Jia B., Homem R.A., Williamson M.S., Gao S.J., Han H.B., Xiang K.F., Sun X.T., Gao X. (2021). Laboratory selection, cross-resistance, risk assessment to lambda-cyhalothrin resistance, and monitoring of insecticide resistance for plant bug *Lygus pratensis* (Hemiptera: Miridae) in farming-pastoral ecotones of Northern China. J. Econ. Entomol..

[9] Gou C.Q., Sun P., Liu D.C., Dilinuer A., Feng H.Z. (2018). Effects of *Lygus pratensis* (Hemiptera: Miridae) infestation on the nutrient contents and protective enzyme activities in host plants. Acta Ecol. Sin..

[10] Liang H.J., Li Y., Sun C.Y., Feng L.K., Wang P.L., Lu Y.H. (2013). The predation of *Lygus pratensis* (L.) to *Aphis gossypii* Glover. J. Environ. Entomol..

[11] Gould F. (1998). Sustainability of transgenic insecticidal cultivars: Integrating pest genetics and ecology. Annu. Rev. Entomol..

[12] Hardee D.D., Bryan W.W. (1997). Influence of *Bacillus thuringiensis* transgenic and nectarless cotton on insect populations with emphasis on the tarnished plant bug (Heteroptera: Miridae). J. Econ. Entomol..

[13] Greene J.K., Turnipseed S.G., Sullivan M.J., Herzog G.A. (1999). Boll damage by southern green stink bug (Hemiptera: Pentatomidae) and tarnished plant bug (Hemiptera: Miridae) caged on transgenic *Bacillus thuringiensis* cotton. J. Econ. Entomol..

[14] Wu K., Li W., Feng H., Guo Y. (2002). Seasonal abundance of the mirids, *Lygus lucorum* and *Adelphocoris* spp. (Hemiptera: Miridae) on Bt cotton in Northern China. Crop. Prot..

[15] Wu K.M. (2007). Environmental impact and risk management strategies of Bt cotton commercialization in China. Chin. J. Agric. Biotechnol..

[16] Xu Y., Wu K.M., Li H.B., Liu J., Ding R.F., Wang F., Ahtam U., Li H.Q., Wang D.M., Chen X.X. (2012). Effects of transgenic Bt+ CpTI cotton on field abundance of non-target pests and predators in Xinjiang, China. J. Integr. Agric..

[17] Lu Y.H., Liang G.M. (2016). Research advance on the succession of insect pest complex in Bt crop ecosystem. Plant Prot..

[18] Lu Y.H., Liang G.M., Zhang Y.J., Yang X.M. (2020). Advances in the management of insect pests of cotton in China since the 21st century. Chin. J. Appl. Entomol..

[19] Ruberson J.R., Williams L.I. (2000). Biological control of *Lygus* spp.: A component of areawide management. Southwest. Entomol..

[20] Lu Y.H., Wu K.M., Jiang Y.Y., Xia B., Li P., Feng H.Q., Wyckhuys K.A.G., Guo Y.Y. (2010). Mirid bug outbreaks in multiple crops correlated with wide-scale adoption of Bt cotton in China. Science.

[21] Greene J.K., Bundy C.S., Roberts P.M., Leonard B.R. (2006). Identification and management of common boll feeding bugs in cotton. Clemson Extension Report.

[22] Zhang Q., Liu Y.Q., Lu Y.H., Wu K.M. (2017). Toxicity and persistence of four kinds of insecticides against *Apolygus lucorum*. China Cotton.

[23] Wang J., Zhao Y., Ray I., Song M. (2016). Transcriptome responses in alfalfa associated with tolerance to intensive animal grazing. Sci. Rep..

[24] Sosa-Gómez D.R., Corrêa-Ferreira B.S., Kraemer B., Pasini A., Husch P.E., Delfino Vieira C.E., Reis Martinez C.B., Negrão Lopes I.O. (2020). Prevalence, damage, management and insecticide resistance of stink bug populations (Hemiptera: Pentatomidae) in commodity crops. Agric. For. Entomol..

[25] Zhao Y.X., Huang J.M., Ni H., Guo D., Yang F.X., Wang X., Wu S.F., Gao C.F. (2020). Susceptibility of fall armyworm, *Spodoptera frugiperda* (J.E. Smith), to eight insecticides in China, with special reference to lambda-cyhalothrin. Pestic. Biochem. Physiol..

[26] Weisenburger D.D. (1993). Human health effects of agrichemical use. Hum. Pathol..

[27] Desneux N., Decourtye A., Delpuech J.M. (2007). The sublethal effects of pesticides on beneficial arthropods. Annu. Rev. Entomol..

[28] Wan N.F., Fu L.W., Dainese M., Kiær L.P., Hu Y.Q., Xin F.F., Goulson D., Woodcock B.A., Vanbergen A.J., Spurgeon D.J. (2025). Pesticides have negative effects on non-target organisms. Nat. Commun..

[29] Li P.F., Zhen Y.X., Gou C.Q., Wu G., Wang L., Feng H.Z. (2023). Effect of five insecticides against *Aphis gossypii* and their safety evaluation on *Hippodamia variegata*. Cotton Sci..

[30] Zhang L., Lyu H.X., Shi D.D., Li X.C., Ma K.S. (2022). Effects of low doses of acetamiprid and afidopyropen on the parasitic function of *Lysiphlebia japonica* (Ashmead). Chin. J. Pestic. Sci..

[31] Begg G.S., Cook S.M., Dye R., Ferrante M., Franck P., Lavigne C., Lövei G.L., Mansion-Vaquie A., Pell J.K., Petit S.A. (2017). functional overview of conservation biological control. Crop Prot..

[32] Wyckhuys K.A.G., Lu Y.H., Morales H., Vazquez L.L., Legaspi J.C., Eliopoulos P.A., Hernandez L.M. (2013). Current status and potential of conservation biological control for agriculture in the developing world. Biol. Control..

[33] Ge F., Ouyang F., Zhao Z.H. (2014). Ecological management of insects based on ecological services at a landscape scale. Chin. J. Appl. Entomol..

[34] Ju Q., Ouyang F., Qiao F., Ge F. (2020). Quantitative evaluation of predation based on molecular analysis of gut-content. Chin. J. Appl. Entomol..

[35] Zhang G.F., Zhu H.K., Huang L., Wang Y.S., Li T., Huang C., Xian X.Q., Xue Y.T., Gui F.R., Li W.X. (2024). Investigation and molecular evaluation of the natural enemies of *Tuta absoluta* (Meyrick) (Lepidoptera: Gelechiidae) in tomato fields in Yunnan province. Chin. J. Biol. Control..

[36] Wang H.Q., Li J., Luan F.G., Zeng J.Y., Ayi B.T., Ma D.Y. (2011). Primary study on main natural enemy resources and control effects of *Erythroneura Apicalis* Nawa in Turpan. Xinjiang Agric. Sci..

[37] Zhang Q., Zhang R.F., Zhang Q.Q., Ji D.Z., Zhou X., Jin L.H. (2021). Functional response and control potential of *Orius sauteri* (Hemiptera: Anthocoridae) on tea thrips (*Dendrothrips minowai* priesner). Insects.

[38] Kobelt A.J., Yen A.L., Kitching M. (2009). Laboratory validation of rubidium marking of herbivorous insects and their predators. Aust. J. Entomol..

[39] Stam P.A., Newsom L.D., Lambremont E.N. (1987). Predation and food as factors affecting survival of *Nezara viridula* (L.) (Hemiptera: Pentatomidae) in a soybean ecosystem. Environ. Entomol..

[40] Zheng Y.X., Li P.F., Li T.L., Wang K.Y., Gou C.Q., Feng H.Z. (2024). Studies on *Lygus pratensis* (Hemiptera: Miridae) flight ability. Insects.

[41] Furlong M.J. (2015). Knowing your enemies: Integrating molecular and ecological methods to assess the impact of arthropod predators on crop pests. Insect Sci..

[42] Jonsson M., Wratten S.D., Landis D.A., Gurr G.M. (2008). Recent advances in conservation biological control of arthropods by arthropods. Biol. Control..

[43] Pompanon F., Deagle B.E., Symondson W.O., Brown D.S., Jarman S.N., Taberlet P. (2012). Who is eating what: Diet assessment using next generation sequencing. Mol. Ecol..

[44] Galan M., Pons J.B., Tournayre O., Pierre É., Leuchtmann M., Pontier D., Charbonnel N. (2018). Metabarcoding for the parallel identification of several hundred predators and their prey: Application to bat species diet analysis. Mol. Ecol. Resour..

[45] Paula D.P., Linard B., Crampton-Platt A., Srivathsan A., Timmermans M.J.N., Sujii E.R., Pires C.S.S., Souza L.M., Andow D.A., Vogler A.P. (2016). Uncovering trophic interactions in arthropod predators through DNA shotgun-sequencing of gut contents. PLoS ONE.

[46] Bai S.X., Zhao J.P., Gou C.Q., Yao C.C., Feng H.Z. (2022). Effects of farmland landscape pattern on adult population dynamics of *Lygus pratensis* in Aral Reclamation Area of Xinjiang. Cotton Sci..

[47] Ganem Z., Ferrante M., Lubin Y., Armiach Steinpress I., Gish M., Sharon R., Harari A.R., Keasar T., Gavish-Regev E. (2023). Effects of natural habitat and season on cursorial spider assemblages in Mediterranean vineyards. Insects.

[48] Waldner T., Traugott M. (2012). DNA-based analysis of regurgitates: A noninvasive approach to examine the diet of invertebrate consumers. Mol. Ecol. Resour..

[49] King R.A., Read D.S., Traugott M., Symondson W.O. (2008). Molecular analysis of predation: A review of best practice for DNA-based approaches. Mol. Ecol..

[50] Folmer O., Black M., Hoeh W., Lutz R., Vrijenhoek R. (1994). DNA primers for amplification of mitochondrial cytochrome c oxidase subunit I from diverse metazoan invertebrates. Mol. Mar. Biol. Biotechnol..

[51] Mengual X., Bot S., Chkhartishvili T., Reimann A., Thormann J., Mark L. (2020). Checklist of hover flies (*Diptera*, *Syrphidae*) of the Republic of Georgia. ZooKeys.

[52] Rome Q., Perrard A., Muller F., Fontaine C., Quilès A., Zuccon D., Villemant C. (2021). Not just honeybees: Predatory habits of *Vespa velutina* (Hymenoptera: Vespidae) in France. Ann. Société Entomol. Fr..

[53] Penney H.D., Hassall C., Skevington J.H., Abbott K.R., Sherratt T.N. (2012). A comparative analysis of the evolution of imperfect mimicry. Nature.

[54] Wirta H., Várkonyi G., Rasmussen C., Kaartinen R., Schmidt N.M., Hebert P., Barták M., Blagoev G., Disney H., Ertl S. (2016). Establishing a community-wide DNA barcode library as a new tool for arctic research. Mol. Ecol. Resour..

[55] Roslin T., Somervuo P., Pentinsaari M., Hebert P.D.N., Agda J., Ahlroth P., Anttonen P., Aspi J., Blagoev G., Blanco S. (2022). A molecular-based identification resource for the arthropods of Finland. Mol. Ecol. Resour..

[56] Jiménez-García E., Andújar C., López H., Emerson B.C. (2023). Towards understanding insect species introduction and establishment: A community-level barcoding approach using island beetles. Mol. Ecol..

[57] Rulik B., Eberle J., Mark L., Thormann J., Jung M., Köhler F., Apfel W., Weigel A., Kopetz A., Köhler J. (2017). Using taxonomic consistency with semi-automated data pre-processing for high-quality DNA barcodes. Methods. Ecol. Evol..

[58] Pentinsaari M., Hebert P.D., Mutanen M. (2014). Barcoding beetles: A regional survey of 1872 species reveals high identification success and unusually deep interspecific divergences. PLoS ONE.

[59] Nattier R., Michel-Salzat A., Almeida L.M., Chifflet-Belle P., Magro A., Salazar K., Kergoat G.J. (2021). Phylogeny and divergence dating of the ladybird beetle tribe Coccinellini Latreille (Coleoptera: Coccinellidae: Coccinellinae). Syst. Entomol..

[60] Hendrich L., Morinière J., Haszprunar G., Hebert P.D., Hausmann A., Köhler F., Balke M. (2015). A comprehensive DNA barcode database for central European beetles with a focus on Germany: Adding more than 3500 identified species to bold. Mol. Ecol. Resour..

[61] Yi P., Yu P., Liu J., Xu H., Liu X. (2018). A DNA barcode reference library of *Neuroptera* (*Insecta*, *Neuropterida*) from Beijing. ZooKeys.

[62] Rossmann S., Dees M.W., Perminow J., Meadow R., Brurberg M.B. (2018). Soft rot *Enterobacteriaceae* are carried by a large range of insect species in potato fields. Appl. Environ. Microbiol..

[63] Blagoev G.A., Dewaard J.R., Ratnasingham S., Dewaard S.L., Lu L.Q., Robertson J., Telfer A.C., Hebert P.D. (2016). Untangling taxonomy: A DNA barcode reference library for Canadian spiders. Mol. Ecol. Resour..

[64] Astrin J.J., Höfer H., Spelda J., Holstein J., Bayer S., Hendrich L., Huber B.A., Kielhorn K.H., Krammer H.J., Lemke M. (2016). Towards a DNA barcode reference database for spiders and harvestmen of Germany. PLoS ONE.

[65] Wang Z.L., Yang X.Q., Wang T.Z., Yu X. (2018). Assessing the effectiveness of mitochondrial COI and 16S rRNA genes for DNA barcoding of farmland spiders in China. Mitochondrial DNA Part A..

[66] Tanikawa A., Shinkai A., Tatsuta H., Miyashita T. (2018). Highly diversified population structure of the spider *Lycosa ishikariana* inhabiting sandy beach habitats. Conserv. Genet..

[67] Macías-Hernández N., Domènech M., Cardoso P., Emerson B.C., Borges P.A.V., Lozano-Fernandez J., Paulo O.S., Vieira A., Enguídanos A., Rigal F. (2020). Building a robust, densely-sampled spider tree of life for ecosystem research. Diversity.

[68] Pan W.J., Fang H.Y., Zhang P., Pan H.C. (2016). The complete mitochondrial genome of striped lynx spider *Oxyopes sertatus* (Araneae: Oxyopidae). Mitochondrial DNA Part A.

[69] Li Q.Q., Li D.Y., Ye H., Liu X.F., Shi W., Cao N., Duan Y.Q. (2011). Using COI gene sequence to barcode two morphologically alike species: The cotton bollworm and the oriental tobacco budworm (Lepidoptera: Noctuidae). Mol. Biol. Rep..

[70] Jelaska L.S., Franjevic D., Jelaska S.D., Symondson W.O.C. (2014). Prey detection in Carabid beetles (Coleoptera: Carabidae) in woodland ecosystems by PCR analysis of gut contents. Eur. J. Entomol..

[71] Macías-Hernández N., Athey K., Tonzo V., Wangensteen O.S., Arnedo M., Harwood J.D. (2018). Molecular gut content analysis of different spider body parts. PLoS ONE.

[72] Panni S., Pizzolotto R. (2018). Fast molecular assay to detect the rate of decay of *Bactrocera oleae* (Diptera: Tephritidae) DNA in *Pterostichus melas* (Coleoptera: Carabidae) gut contents. Appl. Entomol. Zool..

[73] Xiao D., Xu Q.X., Chen X., Du X.Y., Desneux N., Thomine E., Dai H.J., Harwood J.D., Wang S. (2021). Development of a molecular gut-content identification system to identify aphids preyed upon by the natural enemy *Coccinella septempunctata*. Entomol. Gen..

[74] Chapman E.G., Schmidt J.M., Welch K.D., Harwood J.D. (2013). Molecular evidence for dietary selectivity and pest suppression potential in an epigeal spider community in winter wheat. Biol. Control..

[75] Edgar R.C. (2004). Muscle: A multiple sequence alignment method with reduced time and space complexity. BMC Bioinf..

[76] Rozen S., Skaletsky H. (2000). Primer3 on the WWW for general users and for biologist programmers. Methods. Mol. Biol..

[77] Gou C.Q., Sun P., Liu D.C., Dilinuer A., Feng H.Z. (2019). Effects of different host plants on the growth and development of *Lygus pratensis*. J. Envion. Entomol..

[78] Wang G.C., Sun X.L., Huang W.X., Cai X.M., Chen Z.M. (2010). Predatory Response of Penultimate-instar *Xysticus ephippiatus* Simon on 3-day-old Larval of Tea Loopers under Different Temperatures. J. Tea Sci..

[79] Holling C.S. (1959). Some characteristics of simple types of predation and parasitism1. Can. entomol..

[80] Tang Y.T. (2020). Study on Potential of a Novel Natural Enemy Insect *Picromerus lewisi* Scott in Biological Control. Master’s Thesis.

[81] Sun G.L., Cai H.S., Yang Y., Jin S.T., Zhang Q.J., Tang Y., Chen B. (2025). Predation by *Sycanus croceovittatus* on higher instar larvae of *Spodoptera frugiperda* and *Helicoverpa armigera*. Plant Prot..

[82] Wang S.J., Wu J., Zhao Y.N., Li R.X., Zhao D.X. (2020). Functional response of adult *Hierodula patellifera* (Serville, 1839) (Mantodea: Mantidae) to *Tessaratoma papillosa* (Drury) (Hemiptera: Tessaratomidae). Int. J. Trop. Insect Sci..

[83] Tong Y.J., Wu K.M., Gao X.W. (2009). Predation of *Misumenopos tricuspidatus* on Mirids, *Apolygus lucorum* and *Adelphocoris lineolatus*. Chin. J. Biol. Control..

[84] Greenstone M.H., Payton M.E., Weber D.C., Simmons A.M. (2014). The detectability half-life in arthropod predator–prey research: What it is, why we need it, how to measure it, and how to use it. Mol. Ecol..

[85] Quan X.Y., Xia W.S., Liu F.X., Chen J., Peng Y. (2010). Predation of spiders on a new invasive lace bug *Corythucha ciliata* (Say). J. Plant Prot..

[86] Lazzerini G., Camera A., Benedettelli S., Vazzana C. (2007). The role of field margins in agro-biodiversity management at the farm level. Ital. J. Agron..

[87] Schneider S., Widmer F., Jacot K., Kölliker R., Enkerli J. (2012). Spatial distribution of metarhizium clade 1 in agricultural landscapes with arable land and different semi-natural habitats. Appl. Soil Ecol..

[88] Jia J.J., Chen J.Y., Zhang F.P., Niu L.M., Fu Y.G. (2019). Function response of *Neoseiuius californicus* feeding on *Eotetranychus sexmaculatus* at different temperatures. J. Environ. Entomol..

[89] Xu H.Y., Yang N.W., Wang F.H. (2011). Lethal interference between natural enemies in insect community. Acta Ecol. Sin..

[90] Zang L.S., Liu T.X. (2007). Intraguild interactions between an oligophagous predator, *Delphastus catalinae* (Coleoptera: Coccinellidae), and a parasitoid, *Encarsia sophia* (Hymenoptera: Aphelinidae), of *Bemisia tabaci* (Homoptera: Aleyrodidae). Biol. Control.

[91] Collier T.R., Hunter M.S., Kelly S.E. (2007). Heterospecific ovicide influences the outcome of competition between two endoparasitoids, *Encarsia formosa* and *Encarsia luteola*. Ecol. Entomol..

[92] Yin Z., Li J.P., Dong M., Hou Z.R., Sun B.B., Guo X.H. (2017). Research on predation capacity and preference of *Orius sauteri* agains western flower thrips (*Frankliniella occidentalis*), two-spotted spider mite (*Tetranychus urticae*) and peach aphid (*Myzus persicae*). China Plant Prot..

[93] Chen J.H., Zhang T.H., Liu H.M., Yang S., Xu M.F., Guo S.B. (2025). Predation ability and behavior of *Eocanthecona furcellata* to *Ectropis grisescens* larvae. Plant Prot..

